# 2-[4-(Dimethyl­amino)phen­yl]imidazo[4,5-*f*][1,10]phenanthroline sesquihydrate

**DOI:** 10.1107/S1600536808016930

**Published:** 2008-06-07

**Authors:** Gang-Qiang Yin

**Affiliations:** a Department of Chemistry, Guangdong Medical College, Zhanjiang, Guangdong 524023, People’s Republic of China

## Abstract

There are two formula units in the asymmetric unit of the title compound, C_21_H_17_N_5_·1.5H_2_O. The imidazo[4,5-*f*][1,10]phen­an­throline unit is almost coplanar with the benzene ring, the dihedral angles between them being 8.91 (5) and 4.93 (6)° in the two mol­ecules. The crystal structure is stabilized by a series of hydrogen bonds between the water mol­ecules and the N atoms of the imidazophenanthroline groups.

## Related literature

For related literature, see: Sun *et al.* (2007 ▶). For bond-length data, see: Allen *et al.* (1987 ▶).
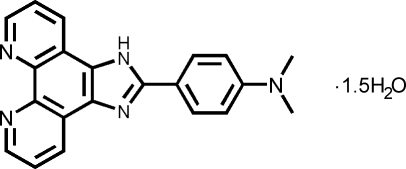


## Experimental

### 

#### Crystal data

C_21_H_17_N_5_·1.5H_2_O
*M* *_r_* = 386.42
Triclinic, 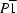

*a* = 11.0503 (9) Å
*b* = 12.6386 (8) Å
*c* = 14.0297 (11) Å
α = 73.685 (9)°
β = 81.909 (10)°
γ = 79.163 (9)°
*V* = 1838.9 (3) Å^3^
*Z* = 4
Mo *K*α radiation
μ = 0.09 mm^−1^
*T* = 293 (2) K
0.45 × 0.35 × 0.30 mm

#### Data collection

Bruker SMART CCD area-detector diffractometer
Absorption correction: multi-scan (*CrystalClear*; Rigaku/MSC, 2000 ▶) *T*_min_ = 0.961, *T*_max_ = 0.974
15313 measured reflections
8974 independent reflections
5671 reflections with *I* > 2σ(*I*)
*R*_int_ = 0.042

#### Refinement

*R*[*F* ^2^ > 2σ(*F* ^2^)] = 0.051
*wR*(*F* ^2^) = 0.138
*S* = 0.96
8974 reflections
520 parameters
H atoms treated by a mixture of independent and constrained refinement
Δρ_max_ = 0.35 e Å^−3^
Δρ_min_ = −0.21 e Å^−3^

### 

Data collection: *CrystalClear* (Rigaku/MSC, 2000 ▶); cell refinement: *CrystalClear*; data reduction: *CrystalClear*; program(s) used to solve structure: *SHELXS97* (Sheldrick, 2008 ▶); program(s) used to refine structure: *SHELXL97* (Sheldrick, 2008 ▶); molecular graphics: *SHELXTL* (Sheldrick, 2008 ▶); software used to prepare material for publication: *SHELXTL*.

## Supplementary Material

Crystal structure: contains datablocks I, global. DOI: 10.1107/S1600536808016930/pk2098sup1.cif

Structure factors: contains datablocks I. DOI: 10.1107/S1600536808016930/pk2098Isup2.hkl

Additional supplementary materials:  crystallographic information; 3D view; checkCIF report

## Figures and Tables

**Table 1:** Hydrogen-bond geometry (Å, °)

*D*—H⋯*A*	*D*—H	H⋯*A*	*D*⋯*A*	*D*—H⋯*A*
O3—H6⋯N8	0.90 (3)	2.01 (3)	2.870 (2)	159 (2)
C18—H18*A*⋯N3	0.93	2.61	2.919 (2)	100
O1—H1⋯O3^i^	0.78 (2)	1.93 (2)	2.711 (2)	176 (2)
O1—H2⋯N4^i^	0.95 (3)	1.95 (3)	2.891 (2)	170 (2)
N9—H7*B*⋯O1^ii^	0.86	1.98	2.820 (2)	166
O2—H3⋯N6^iii^	0.86 (2)	2.34 (2)	3.047 (2)	139 (2)
O2—H3⋯N7^iii^	0.86 (2)	2.15 (2)	2.899 (2)	144 (2)
O2—H4⋯N1^i^	0.92 (3)	2.11 (3)	2.943 (2)	151 (2)
N3—H3*B*⋯O2^iv^	0.86	1.94	2.751 (2)	157
O3—H5⋯N2^i^	0.89 (3)	2.10 (3)	2.969 (2)	163 (2)

Symmetry codes: (i) 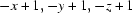; (ii) 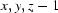; (iii) 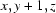; (iv) 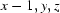.

**Fig. 1.**
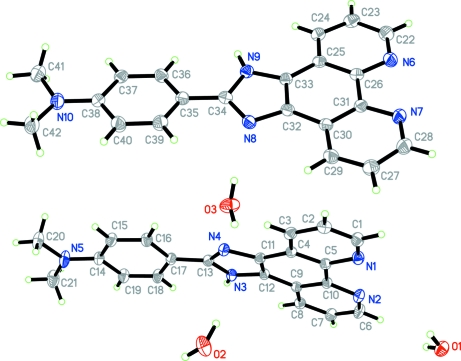
A view of the compound with the atomic numbering scheme. Displacement ellipsoids were drawn at the 30% probability level.

## supplementary crystallographic information

### Comment

1,10-Phenanthroline and its derivatives are commonly used as ligands in metal complexes (e.g. Sun *et al.*, 2007). We report here the structure of the title compound, which was synthesized from [4,5-*f*]1,10-phenanthroline. In this compound, all the bond lengths are within normal ranges (Allen *et al.*, 1987). The asymmetric unit consists of two independent C_21_H_17_N_5_ molecules and three H_2_O molecules (Fig. 1).  Each C_21_H_17_N_5_ molecule consists of imidazo- phenanthroline and phenyl rings. The imidazo[4,5-*f*]1,10-phenanthroline moiety is almost coplanar with the phenyl ring, with dihedral angles between them in each molecule of 8.91 (5)° and 4.93 (6)°.  The three H_2_O molecules link the 2-(4'-Dimethylaminophenyl)imidazo[4,5-*f*]1,10-phenanthroline molecules by hydrogen bonds to the nitrogen atoms of the imidazo-phenantholine ring systems.

### Experimental

1,10-phenanthroline-5,6-dione (1.5 mmol) and dimethylaminobenzaldehyde (1.5 mmol) were dissolved in CH_3_COOH-CH_3_COONH_4_ (1:1) solution(30 ml). The mixture was refluxed for 1.5 h under argon, after cooling, this mixture was diluted with water and neutralized with concentrated aqueous ammonia, immediately resulting a yellow precipitate, which was washed with water, acetone and diethyl ether respectively. Crystals of the title compound were obtained by recrystallization from dichloromethane.

### Refinement

Coordinates of hydrogen atoms bonded to carbon atoms were calculated following the stereochemical rules with C—H distances of 0.93 Å for phenyl and 0.96 Å for methyl groups. The hydrogen atoms were included in the refinement using the riding-model approximation. *U*_iso_(H) were defined as 1.2*U*_eq_ of the parent carbon atoms for phenyl and 1.5*U*_eq_ of the parent carbon atoms for the methyl groups. All H atoms on N atoms were positioned geometrically and refined as riding atoms, with N—H = 0.86 Å and *U*_iso_(H) = 1.2*U*_eq_(N). The H atoms of the waters were located in a Fourier map following isotropic refinement.

### Crystal data

**Table:**

C_21_H_17_N_5_·1.5(H_2_O)	*Z* = 4
*M**_r_* = 386.42	*F*_000_ = 772
Triclinic, *P*1	*D*_x_ = 1.324 Mg m^−^^3^
Hall symbol: -P 1	Mo *K*α radiation λ = 0.71073 Å
*a* = 11.0503 (9) Å	Cell parameters from 4754 reflections
*b* = 12.6386 (8) Å	θ = 1.5–28.3º
*c* = 14.0297 (11) Å	µ = 0.09 mm^−^^1^
α = 73.685 (9)º	*T* = 293 (2) K
β = 81.909 (10)º	Prism, yellow
γ = 79.163 (9)º	0.45 × 0.35 × 0.30 mm
*V* = 1838.9 (3) Å^3^	

### Data collection

**Table:**

Bruker SMART CCD area-detector diffractometer	8974 independent reflections
Radiation source: fine-focus sealed tube	5671 reflections with *I* > 2σ(*I*)
Monochromator: graphite	*R*_int_ = 0.042
*T* = 293(2) K	θ_max_ = 28.3º
φ and ω scans	θ_min_ = 1.9º
Absorption correction: multi-scan(CrystalClear; Rigaku/MSC, 2000)	*h* = −14→14
*T*_min_ = 0.961, *T*_max_ = 0.974	*k* = −16→13
15313 measured reflections	*l* = −18→17

### Refinement

**Table:**

Refinement on *F*^2^	Secondary atom site location: difference Fourier map
Least-squares matrix: full	Hydrogen site location: inferred from neighbouring sites
*R*[*F*^2^ > 2σ(*F*^2^)] = 0.051	H atoms treated by a mixture of independent and constrained refinement
*wR*(*F*^2^) = 0.138	*w* = 1/[σ^2^(*F*_o_^2^) + (0.0641*P*)^2^] where *P* = (*F*_o_^2^ + 2*F*_c_^2^)/3
*S* = 0.96	(Δ/σ)_max_ < 0.001
8974 reflections	Δρ_max_ = 0.35 e Å^−^^3^
520 parameters	Δρ_min_ = −0.21 e Å^−^^3^
Primary atom site location: structure-invariant direct methods	Extinction correction: none

### Special details

**Table:**

Geometry. All e.s.d.'s (except the e.s.d. in the dihedral angle between two l.s. planes) are estimated using the full covariance matrix. The cell e.s.d.'s are taken into account individually in the estimation of e.s.d.'s in distances, angles and torsion angles; correlations between e.s.d.'s in cell parameters are only used when they are defined by crystal symmetry. An approximate (isotropic) treatment of cell e.s.d.'s is used for estimating e.s.d.'s involving l.s. planes.
Refinement. Refinement of *F*^2^ against ALL reflections. The weighted *R*-factor *wR* and goodness of fit *S* are based on *F*^2^, conventional *R*-factors *R* are based on *F*, with *F* set to zero for negative *F*^2^. The threshold expression of *F*^2^ > σ(*F*^2^) is used only for calculating *R*-factors(gt) *etc*. and is not relevant to the choice of reflections for refinement. *R*-factors based on *F*^2^ are statistically about twice as large as those based on *F*, and *R*- factors based on ALL data will be even larger.

### Fractional atomic coordinates and isotropic or equivalent isotropic displacement parameters (Å^2^)

**Table:**

	*x*	*y*	*z*	*U*_iso_*/*U*_eq_	
C1	0.45171 (15)	0.33283 (14)	0.52723 (13)	0.0392 (4)	
H1A	0.4972	0.2657	0.5594	0.047*	
C2	0.50296 (15)	0.39220 (14)	0.43682 (13)	0.0387 (4)	
H2A	0.5814	0.3665	0.4104	0.046*	
C3	0.43558 (15)	0.48961 (14)	0.38724 (12)	0.0348 (4)	
H3A	0.4676	0.5308	0.3261	0.042*	
C4	0.31834 (14)	0.52678 (12)	0.42896 (11)	0.0289 (3)	
C5	0.27578 (14)	0.46184 (13)	0.52285 (12)	0.0301 (3)	
C6	0.01425 (16)	0.46905 (15)	0.70863 (13)	0.0431 (4)	
H6A	−0.0078	0.4281	0.7729	0.052*	
C7	−0.07076 (16)	0.55909 (14)	0.66489 (12)	0.0392 (4)	
H7A	−0.1476	0.5761	0.6985	0.047*	
C8	−0.03942 (15)	0.62191 (14)	0.57195 (12)	0.0347 (4)	
H8A	−0.0940	0.6836	0.5413	0.042*	
C9	0.07598 (14)	0.59251 (12)	0.52306 (11)	0.0291 (3)	
C10	0.15556 (14)	0.49771 (13)	0.57175 (11)	0.0296 (3)	
C11	0.23794 (14)	0.62512 (13)	0.38244 (11)	0.0293 (3)	
C12	0.12222 (14)	0.65243 (12)	0.42739 (11)	0.0297 (3)	
C13	0.15243 (15)	0.77516 (13)	0.28321 (12)	0.0322 (4)	
C14	0.08692 (15)	1.07618 (13)	0.04811 (12)	0.0356 (4)	
C15	0.18800 (15)	0.99284 (14)	0.04114 (12)	0.0395 (4)	
H15A	0.2441	1.0036	−0.0154	0.047*	
C16	0.20693 (16)	0.89581 (14)	0.11507 (13)	0.0386 (4)	
H16A	0.2746	0.8420	0.1068	0.046*	
C17	0.12777 (14)	0.87548 (13)	0.20225 (11)	0.0323 (4)	
C18	0.02535 (16)	0.95707 (14)	0.20844 (13)	0.0404 (4)	
H18A	−0.0310	0.9453	0.2648	0.048*	
C19	0.00444 (16)	1.05446 (14)	0.13433 (13)	0.0409 (4)	
H19A	−0.0654	1.1067	0.1415	0.049*	
C20	0.15919 (19)	1.19547 (17)	−0.11149 (14)	0.0590 (6)	
H20A	0.2375	1.1995	−0.0916	0.089*	
H20B	0.1297	1.2649	−0.1571	0.089*	
H20C	0.1689	1.1361	−0.1435	0.089*	
C21	−0.03024 (19)	1.26246 (16)	−0.01427 (16)	0.0599 (6)	
H21A	−0.0326	1.2769	0.0497	0.090*	
H21B	−0.1068	1.2402	−0.0198	0.090*	
H21C	−0.0185	1.3289	−0.0660	0.090*	
C22	0.54717 (18)	−0.06149 (16)	0.22080 (14)	0.0497 (5)	
H22A	0.5276	−0.1331	0.2468	0.060*	
C23	0.50237 (17)	0.00110 (16)	0.13110 (14)	0.0462 (5)	
H23A	0.4529	−0.0278	0.0993	0.055*	
C24	0.53193 (15)	0.10615 (15)	0.08978 (13)	0.0384 (4)	
H24A	0.5030	0.1497	0.0297	0.046*	
C25	0.60662 (14)	0.14625 (13)	0.14012 (12)	0.0329 (4)	
C26	0.64498 (15)	0.07783 (14)	0.23243 (12)	0.0350 (4)	
C27	0.8597 (2)	0.18442 (16)	0.39974 (15)	0.0555 (5)	
H27A	0.9077	0.2044	0.4393	0.067*	
C28	0.8253 (2)	0.07935 (16)	0.42726 (15)	0.0534 (5)	
H28A	0.8520	0.0299	0.4859	0.064*	
C29	0.82222 (18)	0.25814 (15)	0.31362 (14)	0.0467 (5)	
H29A	0.8445	0.3290	0.2936	0.056*	
C30	0.74997 (15)	0.22578 (13)	0.25602 (12)	0.0353 (4)	
C31	0.71856 (15)	0.11709 (13)	0.28993 (12)	0.0349 (4)	
C32	0.71051 (15)	0.29322 (13)	0.16206 (12)	0.0340 (4)	
C33	0.64546 (14)	0.25301 (13)	0.10671 (12)	0.0323 (4)	
C34	0.68171 (15)	0.42218 (13)	0.02556 (12)	0.0356 (4)	
C35	0.68460 (15)	0.52469 (14)	−0.05363 (12)	0.0365 (4)	
C36	0.61896 (16)	0.54779 (14)	−0.13666 (12)	0.0389 (4)	
H36A	0.5723	0.4959	−0.1418	0.047*	
C37	0.62105 (16)	0.64571 (14)	−0.21179 (12)	0.0380 (4)	
H37A	0.5759	0.6583	−0.2661	0.046*	
C38	0.68999 (16)	0.72604 (14)	−0.20729 (13)	0.0396 (4)	
C39	0.75241 (18)	0.60484 (16)	−0.04965 (14)	0.0478 (5)	
H39A	0.7969	0.5921	0.0051	0.057*	
C40	0.75633 (19)	0.70268 (15)	−0.12370 (14)	0.0501 (5)	
H40A	0.8036	0.7539	−0.1182	0.060*	
C41	0.6379 (2)	0.83872 (18)	−0.37374 (14)	0.0590 (6)	
H41A	0.6566	0.9068	−0.4202	0.089*	
H41B	0.5498	0.8424	−0.3604	0.089*	
H41C	0.6715	0.7769	−0.4016	0.089*	
C42	0.7699 (2)	0.90254 (18)	−0.28036 (16)	0.0651 (6)	
H42A	0.7583	0.9659	−0.3369	0.098*	
H42B	0.8550	0.8677	−0.2830	0.098*	
H42C	0.7486	0.9265	−0.2201	0.098*	
N1	0.34306 (12)	0.36491 (11)	0.57079 (10)	0.0360 (3)	
N2	0.12422 (13)	0.43701 (12)	0.66596 (10)	0.0379 (3)	
N3	0.06820 (12)	0.74814 (11)	0.36341 (9)	0.0311 (3)	
H3B	−0.0049	0.7841	0.3723	0.037*	
N4	0.25682 (12)	0.70252 (11)	0.29184 (10)	0.0328 (3)	
N5	0.07091 (14)	1.17421 (13)	−0.02435 (11)	0.0501 (4)	
N6	0.61562 (14)	−0.02608 (12)	0.27155 (11)	0.0437 (4)	
N7	0.75716 (14)	0.04518 (12)	0.37563 (11)	0.0433 (4)	
N8	0.73203 (13)	0.39979 (11)	0.11132 (10)	0.0375 (3)	
N9	0.62862 (12)	0.33528 (11)	0.01943 (10)	0.0349 (3)	
H7B	0.5917	0.3326	−0.0297	0.042*	
N10	0.69184 (17)	0.82371 (13)	−0.28192 (11)	0.0537 (4)	
O1	0.53530 (14)	0.29356 (11)	0.85874 (10)	0.0434 (3)	
O2	0.81559 (12)	0.80854 (12)	0.38807 (11)	0.0495 (4)	
O3	0.67079 (13)	0.57209 (13)	0.21453 (11)	0.0546 (4)	
H3	0.769 (2)	0.873 (2)	0.3756 (17)	0.075 (7)*	
H1	0.474 (2)	0.3296 (19)	0.8380 (16)	0.064 (7)*	
H5	0.723 (2)	0.5821 (19)	0.2531 (17)	0.078 (8)*	
H2	0.597 (2)	0.297 (2)	0.8037 (19)	0.092 (9)*	
H4	0.774 (2)	0.757 (2)	0.3773 (19)	0.098 (9)*	
H6	0.709 (2)	0.522 (2)	0.181 (2)	0.097 (10)*	

### Atomic displacement parameters (Å^2^)

**Table:**

	*U*^11^	*U*^22^	*U*^33^	*U*^12^	*U*^13^	*U*^23^
C1	0.0366 (9)	0.0371 (9)	0.0415 (10)	0.0016 (8)	−0.0144 (8)	−0.0061 (8)
C2	0.0330 (8)	0.0421 (10)	0.0435 (10)	−0.0035 (7)	−0.0054 (7)	−0.0160 (8)
C3	0.0350 (8)	0.0402 (9)	0.0314 (9)	−0.0090 (7)	−0.0053 (7)	−0.0096 (7)
C4	0.0298 (8)	0.0300 (8)	0.0291 (8)	−0.0070 (6)	−0.0076 (6)	−0.0074 (7)
C5	0.0310 (8)	0.0299 (8)	0.0312 (8)	−0.0075 (7)	−0.0092 (7)	−0.0063 (7)
C6	0.0430 (10)	0.0497 (11)	0.0294 (9)	−0.0084 (9)	−0.0006 (8)	0.0005 (8)
C7	0.0365 (9)	0.0438 (10)	0.0349 (9)	−0.0081 (8)	0.0010 (7)	−0.0072 (8)
C8	0.0342 (8)	0.0344 (9)	0.0361 (9)	−0.0067 (7)	−0.0053 (7)	−0.0086 (7)
C9	0.0311 (8)	0.0281 (8)	0.0304 (8)	−0.0086 (6)	−0.0062 (6)	−0.0070 (7)
C10	0.0324 (8)	0.0300 (8)	0.0272 (8)	−0.0098 (7)	−0.0073 (6)	−0.0036 (7)
C11	0.0312 (8)	0.0315 (8)	0.0265 (8)	−0.0083 (7)	−0.0072 (6)	−0.0051 (7)
C12	0.0328 (8)	0.0281 (8)	0.0288 (8)	−0.0074 (6)	−0.0083 (6)	−0.0042 (7)
C13	0.0340 (8)	0.0325 (8)	0.0309 (9)	−0.0095 (7)	−0.0017 (7)	−0.0074 (7)
C14	0.0340 (8)	0.0333 (9)	0.0347 (9)	−0.0049 (7)	−0.0046 (7)	−0.0010 (7)
C15	0.0372 (9)	0.0408 (10)	0.0330 (9)	−0.0038 (8)	0.0039 (7)	−0.0027 (8)
C16	0.0374 (9)	0.0346 (9)	0.0385 (10)	−0.0019 (7)	−0.0021 (8)	−0.0041 (8)
C17	0.0342 (8)	0.0330 (8)	0.0284 (8)	−0.0085 (7)	−0.0051 (7)	−0.0028 (7)
C18	0.0373 (9)	0.0385 (9)	0.0370 (10)	−0.0047 (8)	0.0057 (7)	−0.0018 (8)
C19	0.0352 (9)	0.0370 (9)	0.0398 (10)	0.0030 (7)	0.0028 (8)	−0.0018 (8)
C20	0.0584 (12)	0.0519 (12)	0.0471 (12)	−0.0040 (10)	0.0041 (10)	0.0115 (10)
C21	0.0555 (12)	0.0444 (11)	0.0599 (13)	0.0072 (10)	0.0013 (10)	0.0054 (10)
C22	0.0594 (12)	0.0454 (11)	0.0507 (12)	−0.0228 (9)	−0.0038 (10)	−0.0139 (9)
C23	0.0457 (10)	0.0566 (12)	0.0457 (11)	−0.0211 (9)	−0.0022 (9)	−0.0208 (9)
C24	0.0355 (9)	0.0452 (10)	0.0369 (10)	−0.0088 (8)	−0.0025 (7)	−0.0133 (8)
C25	0.0293 (8)	0.0360 (9)	0.0346 (9)	−0.0040 (7)	0.0001 (7)	−0.0135 (7)
C26	0.0350 (8)	0.0365 (9)	0.0358 (9)	−0.0049 (7)	−0.0016 (7)	−0.0143 (7)
C27	0.0761 (14)	0.0497 (11)	0.0509 (12)	−0.0159 (11)	−0.0289 (10)	−0.0144 (10)
C28	0.0730 (14)	0.0482 (11)	0.0426 (11)	−0.0135 (10)	−0.0199 (10)	−0.0081 (9)
C29	0.0603 (12)	0.0384 (10)	0.0469 (11)	−0.0114 (9)	−0.0159 (9)	−0.0122 (9)
C30	0.0392 (9)	0.0337 (8)	0.0351 (9)	−0.0038 (7)	−0.0046 (7)	−0.0130 (7)
C31	0.0374 (9)	0.0345 (9)	0.0329 (9)	−0.0017 (7)	−0.0060 (7)	−0.0102 (7)
C32	0.0355 (8)	0.0327 (8)	0.0351 (9)	−0.0025 (7)	−0.0047 (7)	−0.0122 (7)
C33	0.0313 (8)	0.0347 (8)	0.0310 (9)	−0.0009 (7)	−0.0057 (7)	−0.0102 (7)
C34	0.0354 (8)	0.0353 (9)	0.0378 (10)	−0.0042 (7)	−0.0029 (7)	−0.0134 (8)
C35	0.0384 (9)	0.0355 (9)	0.0363 (9)	−0.0048 (7)	−0.0042 (7)	−0.0108 (7)
C36	0.0402 (9)	0.0369 (9)	0.0414 (10)	−0.0084 (8)	−0.0047 (8)	−0.0111 (8)
C37	0.0419 (9)	0.0406 (9)	0.0328 (9)	−0.0046 (8)	−0.0089 (7)	−0.0104 (8)
C38	0.0464 (10)	0.0386 (9)	0.0334 (9)	−0.0074 (8)	0.0007 (8)	−0.0103 (8)
C39	0.0606 (12)	0.0489 (11)	0.0398 (10)	−0.0169 (10)	−0.0183 (9)	−0.0087 (9)
C40	0.0635 (12)	0.0439 (11)	0.0488 (11)	−0.0208 (10)	−0.0141 (10)	−0.0092 (9)
C41	0.0721 (14)	0.0602 (13)	0.0424 (12)	−0.0160 (11)	−0.0035 (10)	−0.0069 (10)
C42	0.0895 (16)	0.0610 (14)	0.0502 (13)	−0.0392 (13)	−0.0047 (12)	−0.0061 (10)
N1	0.0347 (7)	0.0348 (7)	0.0368 (8)	−0.0045 (6)	−0.0093 (6)	−0.0045 (6)
N2	0.0388 (8)	0.0419 (8)	0.0292 (8)	−0.0089 (7)	−0.0055 (6)	−0.0004 (6)
N3	0.0296 (7)	0.0305 (7)	0.0305 (7)	−0.0038 (6)	−0.0040 (6)	−0.0036 (6)
N4	0.0337 (7)	0.0328 (7)	0.0299 (7)	−0.0062 (6)	−0.0047 (6)	−0.0036 (6)
N5	0.0471 (9)	0.0417 (9)	0.0400 (9)	0.0060 (7)	0.0071 (7)	0.0103 (7)
N6	0.0519 (9)	0.0367 (8)	0.0449 (9)	−0.0121 (7)	−0.0070 (7)	−0.0097 (7)
N7	0.0544 (9)	0.0396 (8)	0.0372 (8)	−0.0072 (7)	−0.0163 (7)	−0.0064 (7)
N8	0.0425 (8)	0.0322 (7)	0.0391 (8)	−0.0052 (6)	−0.0077 (6)	−0.0102 (6)
N9	0.0351 (7)	0.0374 (8)	0.0334 (8)	−0.0042 (6)	−0.0076 (6)	−0.0099 (6)
N10	0.0777 (12)	0.0483 (9)	0.0387 (9)	−0.0280 (9)	−0.0114 (8)	−0.0024 (7)
O1	0.0402 (7)	0.0500 (8)	0.0377 (7)	−0.0026 (6)	−0.0087 (6)	−0.0085 (6)
O2	0.0374 (7)	0.0348 (7)	0.0691 (10)	−0.0051 (6)	−0.0072 (6)	−0.0013 (7)
O3	0.0456 (8)	0.0702 (10)	0.0561 (9)	0.0041 (7)	−0.0200 (7)	−0.0309 (8)

### Geometric parameters (Å, °)

**Table:**

C1—N1	1.315 (2)	C23—H23A	0.9300
C1—C2	1.384 (2)	C24—C25	1.400 (2)
C1—H1A	0.9300	C24—H24A	0.9300
C2—C3	1.369 (2)	C25—C26	1.411 (2)
C2—H2A	0.9300	C25—C33	1.425 (2)
C3—C4	1.398 (2)	C26—N6	1.355 (2)
C3—H3A	0.9300	C26—C31	1.456 (2)
C4—C5	1.412 (2)	C27—C29	1.365 (3)
C4—C11	1.433 (2)	C27—C28	1.385 (3)
C5—N1	1.3570 (19)	C27—H27A	0.9300
C5—C10	1.460 (2)	C28—N7	1.315 (2)
C6—N2	1.321 (2)	C28—H28A	0.9300
C6—C7	1.386 (2)	C29—C30	1.396 (2)
C6—H6A	0.9300	C29—H29A	0.9300
C7—C8	1.358 (2)	C30—C31	1.414 (2)
C7—H7A	0.9300	C30—C32	1.430 (2)
C8—C9	1.398 (2)	C31—N7	1.356 (2)
C8—H8A	0.9300	C32—C33	1.376 (2)
C9—C10	1.410 (2)	C32—N8	1.383 (2)
C9—C12	1.420 (2)	C33—N9	1.375 (2)
C10—N2	1.363 (2)	C34—N8	1.332 (2)
C11—C12	1.372 (2)	C34—N9	1.366 (2)
C11—N4	1.3855 (19)	C34—C35	1.453 (2)
C12—N3	1.3763 (19)	C35—C39	1.386 (2)
C13—N4	1.329 (2)	C35—C36	1.391 (2)
C13—N3	1.3630 (19)	C36—C37	1.384 (2)
C13—C17	1.456 (2)	C36—H36A	0.9300
C14—N5	1.364 (2)	C37—C38	1.398 (2)
C14—C15	1.397 (2)	C37—H37A	0.9300
C14—C19	1.402 (2)	C38—N10	1.378 (2)
C15—C16	1.369 (2)	C38—C40	1.402 (3)
C15—H15A	0.9300	C39—C40	1.377 (3)
C16—C17	1.391 (2)	C39—H39A	0.9300
C16—H16A	0.9300	C40—H40A	0.9300
C17—C18	1.391 (2)	C41—N10	1.445 (2)
C18—C19	1.375 (2)	C41—H41A	0.9600
C18—H18A	0.9300	C41—H41B	0.9600
C19—H19A	0.9300	C41—H41C	0.9600
C20—N5	1.448 (2)	C42—N10	1.442 (3)
C20—H20A	0.9600	C42—H42A	0.9600
C20—H20B	0.9600	C42—H42B	0.9600
C20—H20C	0.9600	C42—H42C	0.9600
C21—N5	1.445 (2)	N3—H3B	0.8600
C21—H21A	0.9600	N9—H7B	0.8600
C21—H21B	0.9600	O1—H1	0.78 (2)
C21—H21C	0.9600	O1—H2	0.95 (3)
C22—N6	1.318 (2)	O2—H3	0.86 (2)
C22—C23	1.387 (3)	O2—H4	0.92 (3)
C22—H22A	0.9300	O3—H5	0.89 (3)
C23—C24	1.372 (2)	O3—H6	0.90 (3)
			
N1—C1—C2	124.46 (15)	C26—C25—C33	116.55 (15)
N1—C1—H1A	117.8	N6—C26—C25	122.21 (16)
C2—C1—H1A	117.8	N6—C26—C31	117.10 (15)
C3—C2—C1	118.45 (16)	C25—C26—C31	120.69 (15)
C3—C2—H2A	120.8	C29—C27—C28	118.96 (19)
C1—C2—H2A	120.8	C29—C27—H27A	120.5
C2—C3—C4	119.64 (15)	C28—C27—H27A	120.5
C2—C3—H3A	120.2	N7—C28—C27	124.08 (18)
C4—C3—H3A	120.2	N7—C28—H28A	118.0
C3—C4—C5	117.56 (14)	C27—C28—H28A	118.0
C3—C4—C11	124.56 (14)	C27—C29—C30	119.16 (17)
C5—C4—C11	117.88 (14)	C27—C29—H29A	120.4
N1—C5—C4	122.17 (14)	C30—C29—H29A	120.4
N1—C5—C10	117.56 (14)	C29—C30—C31	118.13 (16)
C4—C5—C10	120.27 (14)	C29—C30—C32	124.34 (16)
N2—C6—C7	125.02 (15)	C31—C30—C32	117.44 (15)
N2—C6—H6A	117.5	N7—C31—C30	121.65 (16)
C7—C6—H6A	117.5	N7—C31—C26	117.60 (15)
C8—C7—C6	118.58 (16)	C30—C31—C26	120.74 (15)
C8—C7—H7A	120.7	C33—C32—N8	110.09 (14)
C6—C7—H7A	120.7	C33—C32—C30	121.00 (15)
C7—C8—C9	119.08 (15)	N8—C32—C30	128.87 (16)
C7—C8—H8A	120.5	N9—C33—C32	106.12 (14)
C9—C8—H8A	120.5	N9—C33—C25	130.44 (15)
C8—C9—C10	118.70 (14)	C32—C33—C25	123.44 (15)
C8—C9—C12	125.07 (14)	N8—C34—N9	111.70 (14)
C10—C9—C12	116.21 (14)	N8—C34—C35	124.89 (16)
N2—C10—C9	121.60 (14)	N9—C34—C35	123.40 (15)
N2—C10—C5	117.57 (14)	C39—C35—C36	116.67 (16)
C9—C10—C5	120.82 (14)	C39—C35—C34	121.45 (16)
C12—C11—N4	110.16 (13)	C36—C35—C34	121.88 (16)
C12—C11—C4	120.34 (14)	C37—C36—C35	121.91 (17)
N4—C11—C4	129.48 (14)	C37—C36—H36A	119.0
C11—C12—N3	106.02 (13)	C35—C36—H36A	119.0
C11—C12—C9	124.25 (14)	C36—C37—C38	121.05 (16)
N3—C12—C9	129.68 (14)	C36—C37—H37A	119.5
N4—C13—N3	111.78 (13)	C38—C37—H37A	119.5
N4—C13—C17	126.34 (14)	N10—C38—C37	121.09 (17)
N3—C13—C17	121.82 (14)	N10—C38—C40	121.86 (17)
N5—C14—C15	121.61 (15)	C37—C38—C40	117.05 (16)
N5—C14—C19	121.95 (15)	C40—C39—C35	122.46 (18)
C15—C14—C19	116.43 (14)	C40—C39—H39A	118.8
C16—C15—C14	121.96 (15)	C35—C39—H39A	118.8
C16—C15—H15A	119.0	C39—C40—C38	120.86 (18)
C14—C15—H15A	119.0	C39—C40—H40A	119.6
C15—C16—C17	121.85 (16)	C38—C40—H40A	119.6
C15—C16—H16A	119.1	N10—C41—H41A	109.5
C17—C16—H16A	119.1	N10—C41—H41B	109.5
C16—C17—C18	116.35 (14)	H41A—C41—H41B	109.5
C16—C17—C13	121.61 (14)	N10—C41—H41C	109.5
C18—C17—C13	122.02 (14)	H41A—C41—H41C	109.5
C19—C18—C17	122.41 (15)	H41B—C41—H41C	109.5
C19—C18—H18A	118.8	N10—C42—H42A	109.5
C17—C18—H18A	118.8	N10—C42—H42B	109.5
C18—C19—C14	120.94 (15)	H42A—C42—H42B	109.5
C18—C19—H19A	119.5	N10—C42—H42C	109.5
C14—C19—H19A	119.5	H42A—C42—H42C	109.5
N5—C20—H20A	109.5	H42B—C42—H42C	109.5
N5—C20—H20B	109.5	C1—N1—C5	117.67 (14)
H20A—C20—H20B	109.5	C6—N2—C10	116.97 (14)
N5—C20—H20C	109.5	C13—N3—C12	107.03 (13)
H20A—C20—H20C	109.5	C13—N3—H3B	126.5
H20B—C20—H20C	109.5	C12—N3—H3B	126.5
N5—C21—H21A	109.5	C13—N4—C11	105.00 (13)
N5—C21—H21B	109.5	C14—N5—C21	121.34 (14)
H21A—C21—H21B	109.5	C14—N5—C20	120.76 (14)
N5—C21—H21C	109.5	C21—N5—C20	117.81 (14)
H21A—C21—H21C	109.5	C22—N6—C26	117.48 (16)
H21B—C21—H21C	109.5	C28—N7—C31	118.01 (16)
N6—C22—C23	124.27 (17)	C34—N8—C32	105.13 (14)
N6—C22—H22A	117.9	C34—N9—C33	106.95 (14)
C23—C22—H22A	117.9	C34—N9—H7B	126.5
C24—C23—C22	119.17 (18)	C33—N9—H7B	126.5
C24—C23—H23A	120.4	C38—N10—C42	120.77 (17)
C22—C23—H23A	120.4	C38—N10—C41	120.65 (16)
C23—C24—C25	118.49 (17)	C42—N10—C41	117.20 (16)
C23—C24—H24A	120.8	H1—O1—H2	107 (2)
C25—C24—H24A	120.8	H3—O2—H4	108 (2)
C24—C25—C26	118.34 (15)	H5—O3—H6	109 (2)
C24—C25—C33	125.09 (15)		
			
N1—C1—C2—C3	−1.8 (3)	N6—C26—C31—C30	−178.01 (14)
C1—C2—C3—C4	0.5 (3)	C25—C26—C31—C30	1.9 (2)
C2—C3—C4—C5	1.3 (2)	C29—C30—C32—C33	176.12 (16)
C2—C3—C4—C11	−177.76 (15)	C31—C30—C32—C33	−0.6 (2)
C3—C4—C5—N1	−1.9 (2)	C29—C30—C32—N8	−1.2 (3)
C11—C4—C5—N1	177.18 (14)	C31—C30—C32—N8	−177.90 (15)
C3—C4—C5—C10	178.31 (14)	N8—C32—C33—N9	1.17 (17)
C11—C4—C5—C10	−2.6 (2)	C30—C32—C33—N9	−176.61 (14)
N2—C6—C7—C8	−1.6 (3)	N8—C32—C33—C25	−178.44 (14)
C6—C7—C8—C9	1.2 (3)	C30—C32—C33—C25	3.8 (2)
C7—C8—C9—C10	0.6 (2)	C24—C25—C33—N9	−5.0 (3)
C7—C8—C9—C12	−177.95 (16)	C26—C25—C33—N9	176.56 (15)
C8—C9—C10—N2	−2.3 (2)	C24—C25—C33—C32	174.52 (15)
C12—C9—C10—N2	176.43 (15)	C26—C25—C33—C32	−3.9 (2)
C8—C9—C10—C5	178.43 (14)	N8—C34—C35—C39	6.5 (3)
C12—C9—C10—C5	−2.9 (2)	N9—C34—C35—C39	−171.90 (15)
N1—C5—C10—N2	5.9 (2)	N8—C34—C35—C36	−172.97 (16)
C4—C5—C10—N2	−174.34 (15)	N9—C34—C35—C36	8.6 (2)
N1—C5—C10—C9	−174.76 (14)	C39—C35—C36—C37	0.1 (3)
C4—C5—C10—C9	5.0 (2)	C34—C35—C36—C37	179.58 (15)
C3—C4—C11—C12	177.28 (15)	C35—C36—C37—C38	0.1 (3)
C5—C4—C11—C12	−1.8 (2)	C36—C37—C38—N10	−179.81 (16)
C3—C4—C11—N4	−1.1 (3)	C36—C37—C38—C40	0.0 (3)
C5—C4—C11—N4	179.86 (16)	C36—C35—C39—C40	−0.4 (3)
N4—C11—C12—N3	0.25 (18)	C34—C35—C39—C40	−179.87 (17)
C4—C11—C12—N3	−178.41 (14)	C35—C39—C40—C38	0.5 (3)
N4—C11—C12—C9	−177.31 (15)	N10—C38—C40—C39	179.53 (18)
C4—C11—C12—C9	4.0 (2)	C37—C38—C40—C39	−0.3 (3)
C8—C9—C12—C11	176.98 (15)	C2—C1—N1—C5	1.2 (3)
C10—C9—C12—C11	−1.6 (2)	C4—C5—N1—C1	0.7 (2)
C8—C9—C12—N3	0.0 (3)	C10—C5—N1—C1	−179.54 (14)
C10—C9—C12—N3	−178.55 (15)	C7—C6—N2—C10	0.0 (3)
N5—C14—C15—C16	177.78 (18)	C9—C10—N2—C6	1.9 (2)
C19—C14—C15—C16	−1.0 (3)	C5—C10—N2—C6	−178.74 (15)
C14—C15—C16—C17	−1.2 (3)	N4—C13—N3—C12	0.15 (18)
C15—C16—C17—C18	2.6 (3)	C17—C13—N3—C12	−177.26 (14)
C15—C16—C17—C13	−176.22 (17)	C11—C12—N3—C13	−0.24 (17)
N4—C13—C17—C16	11.0 (3)	C9—C12—N3—C13	177.13 (16)
N3—C13—C17—C16	−171.95 (15)	N3—C13—N4—C11	0.00 (18)
N4—C13—C17—C18	−167.74 (17)	C17—C13—N4—C11	177.27 (16)
N3—C13—C17—C18	9.3 (3)	C12—C11—N4—C13	−0.15 (18)
C16—C17—C18—C19	−2.0 (3)	C4—C11—N4—C13	178.34 (16)
C13—C17—C18—C19	176.88 (18)	C15—C14—N5—C21	−176.70 (19)
C17—C18—C19—C14	−0.2 (3)	C19—C14—N5—C21	2.0 (3)
N5—C14—C19—C18	−177.12 (18)	C15—C14—N5—C20	−0.2 (3)
C15—C14—C19—C18	1.7 (3)	C19—C14—N5—C20	178.50 (18)
N6—C22—C23—C24	1.4 (3)	C23—C22—N6—C26	−0.8 (3)
C22—C23—C24—C25	0.0 (3)	C25—C26—N6—C22	−1.1 (2)
C23—C24—C25—C26	−1.8 (2)	C31—C26—N6—C22	178.83 (15)
C23—C24—C25—C33	179.79 (15)	C27—C28—N7—C31	0.2 (3)
C24—C25—C26—N6	2.4 (2)	C30—C31—N7—C28	0.1 (3)
C33—C25—C26—N6	−179.01 (14)	C26—C31—N7—C28	178.98 (16)
C24—C25—C26—C31	−177.51 (14)	N9—C34—N8—C32	0.32 (18)
C33—C25—C26—C31	1.0 (2)	C35—C34—N8—C32	−178.26 (15)
C29—C27—C28—N7	−0.4 (3)	C33—C32—N8—C34	−0.93 (17)
C28—C27—C29—C30	0.2 (3)	C30—C32—N8—C34	176.63 (16)
C27—C29—C30—C31	0.1 (3)	N8—C34—N9—C33	0.40 (18)
C27—C29—C30—C32	−176.62 (17)	C35—C34—N9—C33	179.00 (14)
C29—C30—C31—N7	−0.2 (2)	C32—C33—N9—C34	−0.94 (16)
C32—C30—C31—N7	176.68 (15)	C25—C33—N9—C34	178.63 (16)
C29—C30—C31—C26	−179.08 (15)	C37—C38—N10—C42	−175.56 (18)
C32—C30—C31—C26	−2.2 (2)	C40—C38—N10—C42	4.6 (3)
N6—C26—C31—N7	3.1 (2)	C37—C38—N10—C41	−9.3 (3)
C25—C26—C31—N7	−176.97 (15)	C40—C38—N10—C41	170.87 (18)

### Hydrogen-bond geometry (Å, °)

**Table:**

*D*—H···*A*	*D*—H	H···*A*	*D*···*A*	*D*—H···*A*
O3—H6···N8	0.90 (3)	2.01 (3)	2.870 (2)	159 (2)
C18—H18A···N3	0.93	2.61	2.919 (2)	100
O1—H1···O3^i^	0.78 (2)	1.93 (2)	2.711 (2)	176 (2)
O1—H2···N4^i^	0.95 (3)	1.95 (3)	2.891 (2)	170 (2)
N9—H7B···O1^ii^	0.86	1.98	2.820 (2)	166
O2—H3···N6^iii^	0.86 (2)	2.34 (2)	3.047 (2)	139 (2)
O2—H3···N7^iii^	0.86 (2)	2.15 (2)	2.899 (2)	144 (2)
O2—H4···N1^i^	0.92 (3)	2.11 (3)	2.943 (2)	151 (2)
N3—H3B···O2^iv^	0.86	1.94	2.751 (2)	157
O3—H5···N2^i^	0.89 (3)	2.10 (3)	2.969 (2)	163 (2)

Symmetry codes: (i) −*x*+1, −*y*+1, −*z*+1; (ii) *x*, *y*, *z*−1; (iii) *x*, *y*+1, *z*; (iv) *x*−1, *y*, *z*.
